# Evolution and transmission of a conjugative plasmid encoding both ciprofloxacin and ceftriaxone resistance in *Salmonella*

**DOI:** 10.1080/22221751.2019.1585965

**Published:** 2019-03-21

**Authors:** Kaichao Chen, Edward Wai Chi Chan, Sheng Chen

**Affiliations:** a Shenzhen Key Lab for Food Biological Safety Control, Food Safety and Technology Research Center, Hong Kong PolyU Shen Zhen Research Institute, Shenzhen, People’s Republic of China; b Department of Applied Biology and Chemical Technology, State Key Lab of Chirosciences, The Hong Kong Polytechnic University, Kowloon, Hong Kong

**Keywords:** Salmonella, ciprofloxacin resistance, ceftriaxone resistance, PMQR genes, conjugative plasmid

## Abstract

Ceftriaxone and ciprofloxacin are the drugs of choice in treatment of invasive *Salmonella* infections. This study discovered a novel type of plasmid, pSa44-CIP-CRO, which was recovered from a *S.* London strain isolated from meat product and comprised genetic determinants that encoded resistance to both ciprofloxacin and ceftriaxone. This plasmid could be resolved into two daughter plasmids and co-exist with such daughter plasmids in a dynamic form in *Salmonella*; yet it was only present as a single plasmid in *Escherichia coli*. One daughter plasmid, pSa44-CRO, was found to carry the *bla*_CTX-M-130_ gene, which encodes resistance to ceftriaxone, whereas the other plasmid, pSa44-CIP, carried multiple PMQR genes such as *qnrB6-aac(6’)-Ib-cr*, which mediated resistance to ciprofloxacin. These two daughter plasmids could be integrated into one single plasmid through *ISPa40* mediated homologous recombination. Mouse infection and treatment experiments showed that carriage of plasmid, pSa44-CIP-CRO by *S. typhimurium* led to the impairment of treatment by ciprofloxacin or cefitiofur, a veterinary drug with similar properties as ceftriaxone. In conclusion, dissemination of such conjugative plasmids impairs current choices of treatment for life-threatening *Salmonella* infection and hence constitutes a serious public health threat.

## Introduction

*Salmonella* is a leading cause of food-borne illness worldwide [1]. Intestinal salmonellosis is usually self-limiting and resolves in five to seven days without antibiotic treatment. However, bacteremia occurs in 3–10 percent of culture-confirmed cases and is particularly common among patients at the extremes of age and those who are immunocompromised. When infection spreads beyond the intestinal tract, appropriate antimicrobial therapy (e.g. ciprofloxacin in adults and ceftriaxone in children) can be lifesaving [2]. Multidrug resistance in *Salmonella* such as the ACSSuT resistance type of *S. typhimurium* DT104, which has been documented since 1980 [3–6], was thought to originate as a result of the routine practice of giving antimicrobial agents to domestic livestock as a means of preventing and treating diseases, as well as promoting growth. In recent years, the increasing use of fluoroquinolone and cephalosporins in livestock for control and treatment of infectious diseases have caused a steady increase in the incidence of resistance to these two types of antibiotics. Resistance to ceftriaxone in *Salmonella* appears to be slowly increasing, reaching a rate of around 3∼4%, but remained stable in the past few years [7,8]. At the same time, the rate of resistance to ciprofloxacin has increased dramatically in both clinical and food isolates in various countries in recent years, particularly in China and the adjacent areas [9].

Resistance to ceftriaxone is normally mediated by the production of extended-spectrum β-lactamases (ESBLs), the encoding genetic elements of which may be located in plasmids or the chromosome. Among the range of antibiotic-resistant Gram-negative bacteria pathogens known to date, organisms producing CTX-M-type ESBLs pose a particularly serious public health threat worldwide [10]. Often, these enzymes are responsible for therapy failure because of their ability to mediate multidrug resistance. Ciprofloxacin resistance is mainly attributed to double mutations in the *gyrA* gene and a single mutation in the *parC* gene in *Salmonella* [11,12]. Efflux pumps and the presence of plasmid-mediated quinolone resistance (PMQR) determinants have also been regarded as contributive factors of development of low level resistance to nalidixic acid. At least three types of PMQR elements have been reported to date: (i) the Qnr types, which are pentapeptide repeat proteins that bind to DNA gyrase by mimicking double stranded DNA, preventing the binding of fluoroquinolones to gyrase, (ii) the *Aac(6’)-Ib-cr*, a modified aminoglycoside acetyltransferase that hydrolyses fluoroquinolones and (iii) the efflux pumps QepA and OqxAB. PMQR elements were first detected in *Salmonella* in 2009 and have since been increasingly reported [13–18], in particular the newly discovered mobile efflux gene, *oqxAB.* The *oqxAB* and *aac(6’)-Ib-cr* genes often co-exist in the same strain, and may be associated with an increase in incidence of ciprofloxacin resistance in clinical *Salmonella* strains in recent years [19]. Other PMQR genes have also been increasingly reported in *Salmonella* [20]. As a result, a large proportion of ciprofloxacin-resistant *Salmonella* currently harboured only a single mutation or even no mutation in the *gyrA* gene or other target genes, due to carriage of one or more PMQR genes [21]. These *Salmonella* isolates were often found to carry multiple PMQR genes assembled in their chromosome or in non-conjugative plasmids, resulting in rapid development of ciprofloxacin resistance. In this study, we report for the first time the emergence of a type of conjugative plasmid encoding resistance to both ceftriaxone and ciprofloxacin. Transmission of these plasmids in *Salmonella* would significantly hinder the choices of antimicrobial agents in the treatment of life-threatening *Salmonella* infections.

## Results

A total of 157 non-repeated *Salmonella* isolates that were recovered from food samples were reported in our previous study [22]. This study focused on investigation of the mechanisms underlying resistance to both ciprofloxacin and ceftriaxone. Among these 157 isolates, 8% (12/157) and 35% (55/157) were found to be resistant to ceftriaxone and ciprofloxacin, respectively. Three strains, Sa44, Sa54 and Sa115, which belonged to *S.* London, *S.* Indiana and *S.* Albany respectively, were resistant to both ciprofloxacin and ceftriaxone. Conjugation experiments showed that ceftriaxone and ciprofloxacin resistance phenotype of Sa44 could be transferred to *E. coli* J53, while those of other two strains could not. Sequencing of the *gyrA* and *parC* of Sa44 indicated that there was no mutation observed in these two genes. Strain Sa44 was therefore subjected to further study to investigate the mechanisms of its conjugative ciprofloxacin and ceftriaxone resistance.

## Ciprofloxacin and ceftriaxone resistance mediated by conjugative plasmids in *Salmonella*

**Figure 1.**
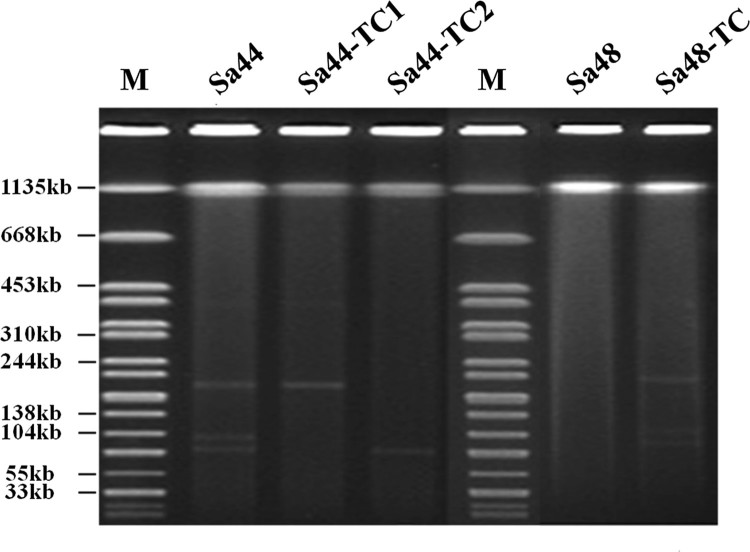
S1-PFGE of ciprofloxacin-resistant *Salmonella* strain Sa44, Sa48 and their corresponding transconjugant*s.* S1-PFGE patterns of *Salmonella* isolates and their relevant conjugants.

*Salmonella* isolate Sa44 was found to carry the *bla*_CTX-M-130_ gene, which was probably responsible for the ceftriaxone resistance phenotype of this strain. It contained no mutation in *gyrA* and *parC*, but carried two PMQR genes, *qnrS* and *aac(6’)-Ib-cr*. Interestingly, conjugation experiments showed that ceftriaxone and ciprofloxacin resistance phenotypes could be transferred to *E. coli* J53 when selected in different types of selective plates. Sa44-TC1 was the transconjugant selected by ciprofloxacin/sodium azide and Sa44-TC2 was the transconjgant selected by ceftriaxone /sodium azide. Antimicrobial susceptibilities were determined for these transconjugants, with results showing that Sa44-TC2 exhibited resistance to cephalosporins, while Sa44-TC1 exhibited resistance to both ciprofloxacin and cephalosporins, suggesting that resistance determinants were encoded on conjugative plasmids (Table 1). Sa44 exhibited MICs of 4 and >16 µg/ml for ciprofloxacin and ceftriaxone, respectively, whereas Sa44-TC1 exhibited MIC of 1 and >16 µg/ml respectively for these two drugs (Table 1). S1-PFGE was performed on this *Salmonella* strain and the corresponding transconjugants. Three plasmids with sizes of ∼200, ∼110 and ∼90 kb were found to be harboured by Sa44, yet only the ∼200 kb plasmid was found in Sa44-TC1 and one ∼90 kb plasmid was detectable in Sa44-TC2 (Figure 1). Interestingly, when the ∼200 kb conjugative plasmid from Sa44-TC1 was transferred to a ciprofloxacin-susceptible (Cip^S^) isogenic *Salmonella* strain, namely *S.* London Sa48, it generated the *Salmonella* transconjugant Sa48-TC. The transconjugant had a CIP MIC level of 4 µg/ml, which approached to that of the parent *Salmonella* strain Sa44, suggesting that this conjugative plasmid could mediate slightly higher MICs in *Salmonella* strains than in *E. coli* (Table 1). S1-PFGE analysis of this transconjugant of *Salmonella* Sa48 depicted the presence of three plasmids with similar sizes as those detected in Sa44 even though only one ∼200 kb plasmid from Sa44-TC1 could be conjugated to Sa48 (Figure 1).

**Table 1.** Genetic and phenotypic characteristics of ceftriaxone and ciprofloxacin-resistant *Salmonella* strains and their corresponding transconjugants.

Strain ID	Species	ESBL/PMQR genes	Plasmids(∼kb)	Mutation	MIC (µg/ml)													
					AZI	AMK	CTX	CIP	KAN	OLA	STR	CRO	TET	CHL	NAL	MRP	AMP	STX
Sa44	*S. London*	*qnrB6-aac(6’)-Ib-cr bla_CTX-M130_*	202, 108 and 90	-	>32	4	>16	4	64	16	>128	>16	>32	>64	16	0.03	>64	>32
Sa44-TC1	*E. coli J53*	*qnrB6-aac(6’)-Ib-cr bla_CTX-M130_*	202, 108 and 90	-	1	2	>16	1	16	8	>128	>16	>32	2	16	0.03	>64	>32
Sa44-TC2	*E. coli J53*	*bla_CTX-M130_*	90	-	1	≤0.5	16	0.015	≤0.5	4	2	16	0.5	2	2	0.03	>64	4
J53	*E. coli*	*-*	-	-	1	≤0.5	≤0.015	0.015	≤0.5	4	2	≤0.015	0.5	1	2	0.03	2	4
Sa48	*S. London*	*-*	-	-	2	1	0.03	0.06	8	16	>128	0.03	>32	64	8	0.03	16	8
Sa48-TC	*S. London*	*qnrB6-aac(6’)-Ib-cr bla_CTX-M130_*	202, 108 and 90	-	2	4	>16	4	>128	32	16	>16	16	2	8	0.03	>64	16

Notes: ND, not determined; ESBL, extended spectrum β-lactamase; PMQR, plasmid mediated quinolone resistance AMK, amikacin; AZI, azithromycin*;* CTX, cefotaxime; CIP, ciprofloxacin; KAN, kanamycin; OLA, olaquidox; STR, streptomycin; CRO, ceftriaxone; TET, tetracycline; CHL, chloramphenicol; NAL, nalidixic acid; AMP, ampicillin; MRP, meropenem; SXT, trimethoprim/sulfamethoxazole. Sa48-TC refers to *Salmonella* conjugant generated through conjugation of Sa44-TC1 to *Salmonella* strain, Sa48, to conjugate the conjugative plasmid back to *Salmonella*.

## Genetic basis of transmission of ciprofloxacin and ceftriaxone resistance in *Salmonella* Sa44

**Figure 2.**
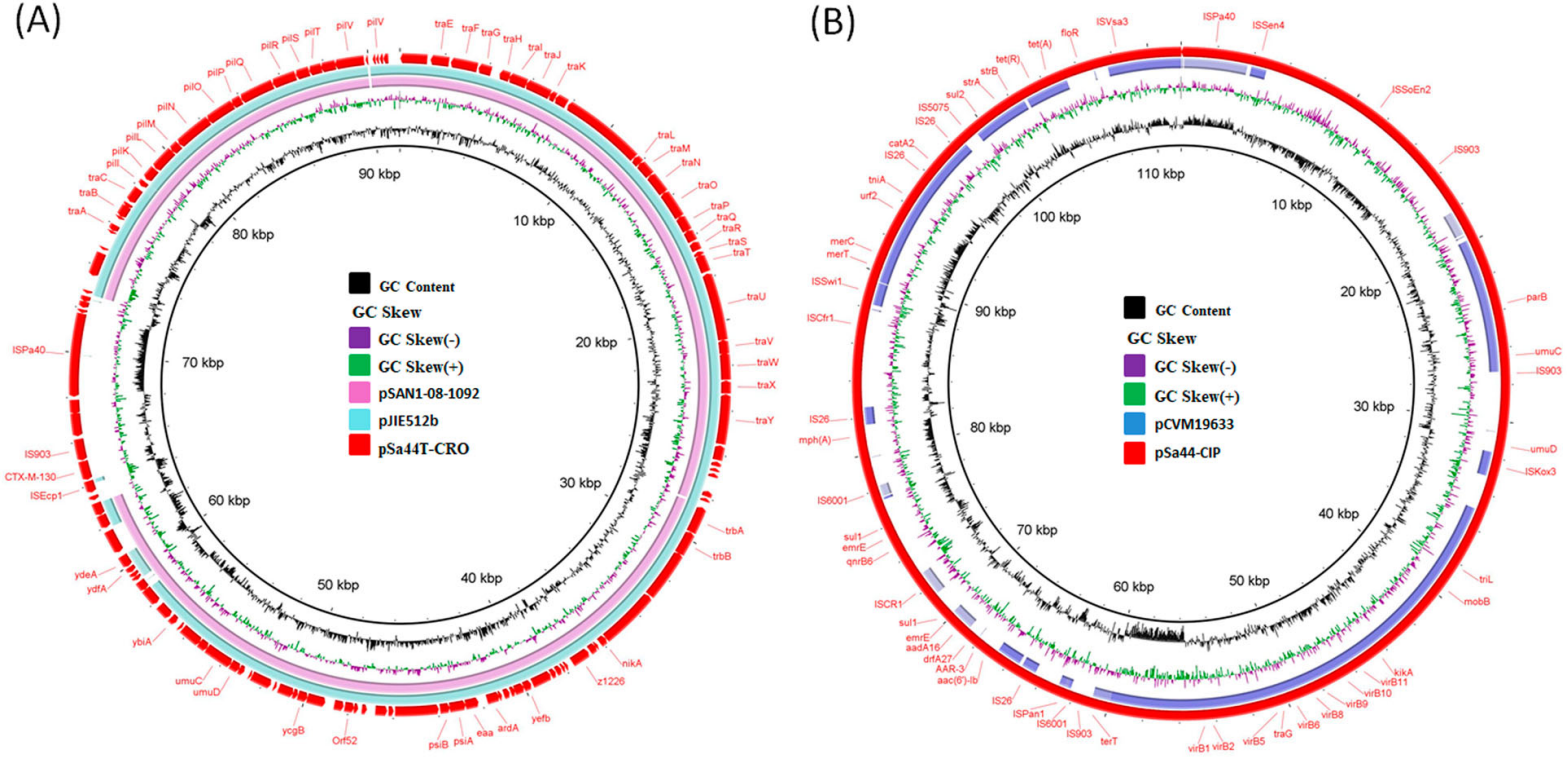
Circular alignment of plasmid pSa44-CRO and pSa44-CIP recovered from *Salmonella* (A) The red circle denotes the plasmid pSa44-CRO that is used as a reference and key genetic loci in this plasmid are labelled. The light blue and pink circle, respectively, represent pSAN1-08-1092(85,439 bp: CP019996.1) and pJIE512b (92,339 bp: NC_025198) in the NCBI database. (B) The light pink circle represents plasmid pCVM19633 (100,227 bp, CP001125) and the outmost circle in red colour represents plasmid pSa44-CIP. Plasmid sequence was generated through the combination of both Illumina and PacBio sequencing data.

To gain further understanding of the genetic features of these conjugative plasmids, complete sequences of the plasmids recovered from Sa44 and its transconjugants were obtained. Three plasmids with complete sequences were resolved in Sa44. The first plasmid, designated pSa44-CRO, had a size of 91,411 bp with GC content of 50.2% and carried 123 CDs. This plasmid was found to belong to the Incl1 incompatibility type and carry mainly IncI1 genes encoding IncI1 conjugative transfer proteins for conjugative functions and a transposition unit, IS*Ecp1*-*bla*_CTX-M130_-IS*903*, which encodes resistance to cephalosporins (Figure 2(a)). BLAST analysis showed that this type of plasmid had previously been reported in *E. coli* and *Salmonella*, with pSAN1-08-1092(85,439 bp: CP019996.1) isolated from an *S. anatum* strain in human stools in the United States, and pJIE512b (92,339 bp: NC_025198) isolated from a clinical *E. coli* strain in Australia, exhibiting the highest degree of sequence homology (Figure 2(a)). In contrast to the absence of any resistance genes in these plasmids, pSa44-CRO carried an IS*Ecp1-bla*_CTX-M13*0*_*-*IS*903* transposition unit.

**Figure 3.**
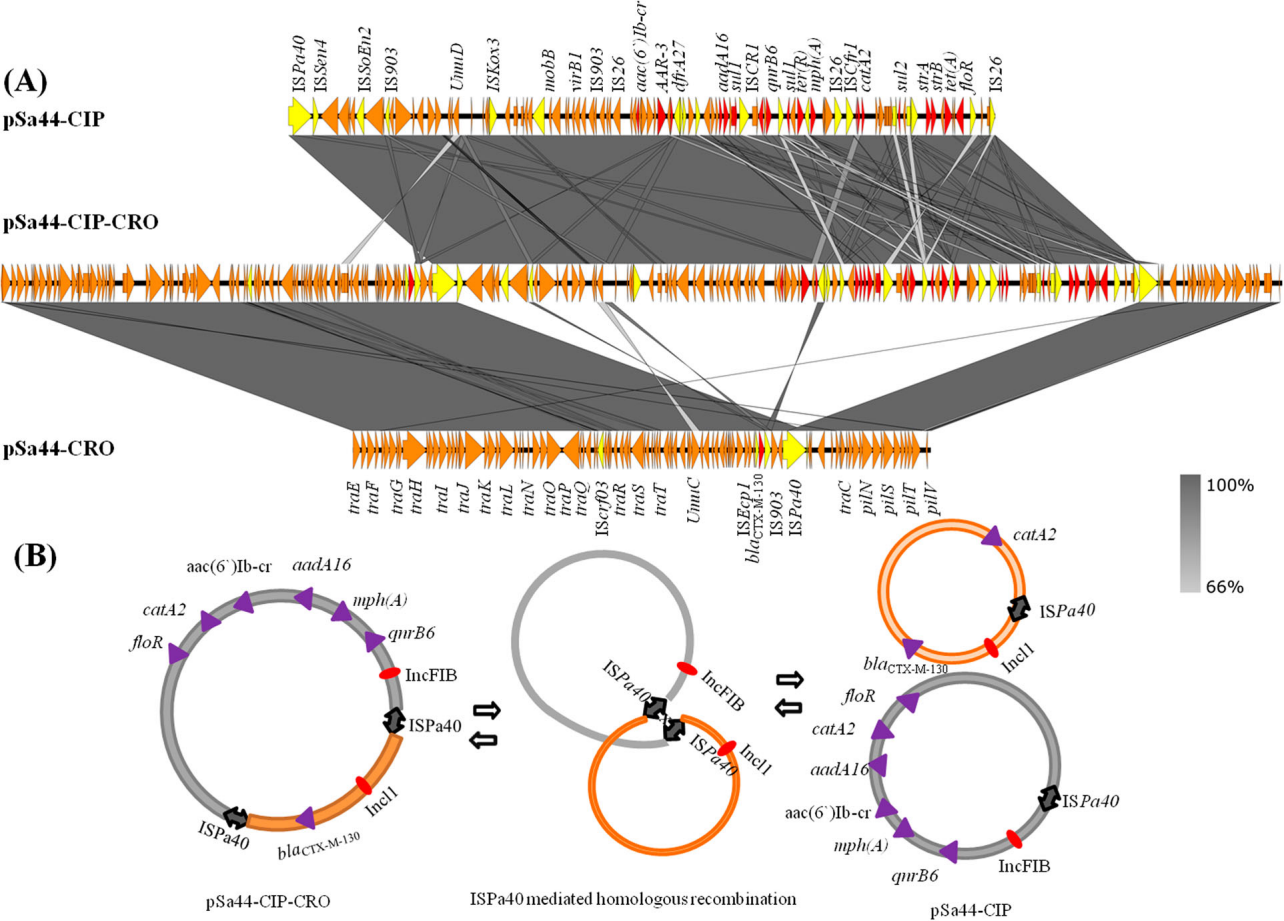
Dynamic presence of hybrid plasmid and daughter plasmids in *Salmonella* with stain Sa44. (A) Alignment of plasmid recovered form strain Sa44 using Easyfig. Plasmids pSa44- CRO (91,411 bp), pSa44-CIP (108,115 bp) and pSa44-CIP-CRO (202,750 bp) were aligned. Key genetic loci in two daughter plasmid were labelled. Plasmid sequence was generated through combination of both Illumina and PacBio sequencing data. Red denotes the resistance genes and yellow denotes ISs (B) Proposed IS element-mediated plasmid fusion through homologous recombination in *Salmonella* Sa44. pSa44-CRO acted as target plasmid, which attacked the hot spot in the donor plasmid, pSa-44-CIP to facilitate plasmid fusion.

The second plasmid, designated pSa44-CIP, was 108,115 bp in size with a GC content of 54.4% and carried 150CDs. It belonged to the IncFIB type and was shown to carry two PMQR genes, *qnrB1*-*aac(6’)-Ib-cr*. BLAST analysis showed that this plasmid has not been reported previously. The only plasmid in GenBank that exhibited homology with it was pCVM19633_110, which was isolated from a *S.* Schwarzengrund strain, CVM19633. This plasmid exhibited 99% similarity with pCVM19633-110, but only 64% coverage. The third and largest plasmid was found to be 202,750 kb in size and belong to IncFIB / Incl1 plasmid family. It exhibited 52.7% GC content and contained 274 CDs. Due to its ability to mediate ciprofloxacin and ceftriaxone resistance, it was designated as pSa44-CIP-CRO. Sequence analysis indicated that this plasmid was the fusion plasmid of pSa44-CRO and pSa44-CIP (Figure 3(a)). Alignment of pSa44-CIP and pSa44-CRO with pSa44-CIP-CRO showed that these plasmids shared a common IS element, IS*Pa40*, at the junction of two daughter plasmids, which allowed us to propose the potential mechanisms of plasmid fusion and resolution. IS*Pa40* present in both daughter plasmids could be used as homologous sequences to form acointegrate between two plasmids. Similarly, daughter plasmids in the cointegrate could be excised through this homologus sequence as shown in Figure 3(b). These three plasmids existed in a dynamic form in *Salmonella*, with both single and fusion plasmids being detectable simultaneously (Figure 1). However, the fusion plasmid pSa44-CIP-CRO seemed to be more stable in *E. coli* and was the only plasmid detectable in *E. coli* strain J53, Sa44-TC1. When pSa44-CIP-CRO was conjugated back into Sa48, three forms of the plasmid including the single form and fusion forms, were again detectable, further confirming the dynamic nature of these plasmids in *Salmonella* (Figure 1).

## Ciprofloxacin and ceftriaxone resistance-encoding conjugative plasmid impaired efficacy of treatment

To test the effect of plasmid pSa44-CIP-CRO on the effectiveness of antimicrobial treatment, the conjugation experiment was performed to transfer pSa44-CIP-CRO from S44-TC1 to strain *S. typhimurium* PY1, producing the pSa44-CIP-CRO-bearing *Salmonella* strain PY1-TC*.* The rationale of using the PY1 strain are (1) PY1 exhibited a higher level of virulence than Sa44 and was more suitable for mouse infection experiments, and (2) PY1 belonged to serotype *S.* Typhimurium, clinically important serotypes. Treatment of PY1-infected mice with ciprofloxacin could significantly reduce the lethal effect of PY1 infection, with a survival rate of 4/5 (80%) recorded at 72 hours, whereas in the other three groups such as the saline treatment groups for PY1 and PY1-TC infection as well as ciprofloxacin treatment group for PY1-TC, 5/5 (100%) mortality at 60 hours was recorded, suggesting that carriage of pS44-CIP-CRO in *S. typhimurium* could render ciprofloxacin treatment ineffective. Similarly, ceftiofur treatment was only effective on mice infected with PY1 (0% mortality) but not PY1-TC (100% mortality) at 36 hours, confirming that carriage of pS44-CIP-CRO could also impair the effectiveness of ceftiofur in the treatment of *Salmonella* infection.

## Discussion

Incidence of resistance to almost all classes of antibiotics including the last resort antibiotics such as carbapenem and colistin has increased sharply worldwide as a result of the emergence of novel antimicrobial resistance genes as well as fast dissemination of mobile elements and plasmids carrying such genes [23]. Upon years of accumulation and evolution of these genetic building blocks in the bacterial communities, assembly of various resistance genes in different formats of mobile elements results in continuous emergence of multidrug resistance-encoding plasmids of novel structures. The advent of high throughput sequencing technologies enabled us to depict the evolution events underlying emergence and transmission of plasmids harbouring resistance genes.

Bacterial resistance to fluoroquinolones such as ciprofloxacin was historically known to be mediated by target gene mutations. Ciprofloxacin resistance in *Salmonella* has previously been attributed exclusively to double *gyrA* mutation and a single *parC* mutation [24]. Due to the low rate of occurrence of *gyrA* double mutations, ciprofloxacin resistance in *Salmonella* remained rare before this period. In 2005, other mechanisms of quinolone resistance such as those due to acquisition of PMQR genes was first reported in *Salmonella*, while these PMQR genes could only mediate quinolone resistance but not resistance to fluoroquinolone such as ciprofloxacin [19,25,26]. In recent years, incidence of ciprofloxacin resistance in *Salmonella* increased sharply in certain parts of the world such as China, reaching 30∼40% for certain serotypes [20,27]. These newly emerged ciprofloxacin-resistant *Salmonella* strains exhibit CIP MIC between 1 and 16 µg/ml, and often harbour a single *gyrA* mutation, along with PMQR genes. Alternatively, some of such strains harbour multiple PMQR genes without any target gene mutations. The resistance phenotype of such strains is therefore presumably due to the synergistic effects of target mutation and a PMQR gene, or that of multiple PMQR elements that act on fluoroquinolones via the mechanisms of enzymatic inactivation, drug efflux, and competitive inhibition of drug binding, producing a high-level quinolone resistance phenotype in the absence of target gene mutations. Recently, we reported the first type of conjugative plasmid that encoded ciprofloxacin resistance phenotype in *Salmonella* [22].

This study identified the first type of conjugative plasmid that encodes resistance to both ciprofloxacin and ceftriaxone. The formation of this plasmid was due to the fusion of two different types of plasmids, each of which comprised a resistance determinant for ciprofloxacin or ceftriaxone respectively. The resulting hybrid plasmids were proven to be transferrable from *Salmonella* to *E. coli* and vise verse without affecting the resistance phenotype. The phenomenon of plasmid fusion has been reported previously [28], in which fusion of IncFIB and IncHI2 plasmids in *Salmonella* strains enabled the resulting fusion product to be transferrable to *E. coli* recipient strains. It appears that plasmid fusions commonly occurs in bacteria in clinical isolation [29], but mechanisms governing the fusion process could not be depicted prior to availability of plasmid sequencing technology [30]. Likewise, conjugative plasmid that facilitates transmission of small non-conjugative IncQ types of plasmids through formation of a conjugation pilus, rather than direct fusion with the IncQ plasmid, was previously reported [31]. Fusion between conjugative plasmids had also been reported, yet our work is the first to discover the dynamic presence of fusion plasmid and the daughter plasmids in a bacterial strain; the underlying biological significance of this phenomenon needs further investigation [32].

In summary, this study identified a novel type of conjugative plasmid that encoded resistance to both ciprofloxacin and ceftriaxone. The emergence of such multidrug resistance-encoding conjugative plasmids among *Salmonella* may have serious impact on global infection control effort since cephalosporins and fluoroquinolones are the key choice of treatment for *Salmonella* infections. New strategies must be urgently devised to halt global transmission of these plasmids among *Salmonella* and other *Enterobacteriaceae* species, as well as organisms which have already acquired such plasmids.

## Materials and methods

### Antimicrobial susceptibility testing

Susceptibility to 14 antimicrobials as listed in Table 1 was determined using the agar dilution method according to the Clinical and Laboratory Standards Institute (CLSI) guidelines [33]. *E. coli* strain ATCC 25922 was used as quality control.

### Conjugation experiments

The transmission of PMQR genes were assessed by performing the conjugation experiment using the filter mating method as previously described [21]. Ciprofloxacin-resistant *Salmonella* strains were used as donor strain and sodium azide-resistant *E. coli* J53 was used as the recipient strains. Ciprofloxacin-resistant transconjugants were selected on EMB agar containing sodium azide (100 µg/ml) and ciprofloxacin (0.5 µg/ml). Cefatriaxone-resistant transconjugants were selected on EMB agar containing sodium azide (100 µg/ml) and ceftriaxone (2 µg/ml).

### Plasmid typing by S1-PFGE

*Salmonella* isolates and their relevant transconjugants were examined by S1-PFGE to determine the size of large plasmids. Briefly, agarose-embedded DNA was digested with S1 nuclease (New England Bio-Lab) at 37°C for 15 min. The linearized digestion products were separated by electrophoresis in 0.5 Tris-borate-EDTA buffer at 14°C for 18 h using a Chef Mapper electrophoresis system (Bio-Rad, Hercules, CA) with pulse times of 2.16–63.8 S. Xba1-PFGE of Salmonella strain H9812 was used as DNA size marker. The gels were stained with GelRed, and DNA bands were visualized with UV transillumination (Bio-Rad).

### Plasmid sequencing and bioinformatics analyses

Plasmid sequencing was conducted on an Illumina platform and a PacBio RSII single-molecule real-time (SMRT) sequencing platform. The Illumina paired-end libraries were constructed with the NEBNext Ultra DNA Library Prep Kit for Illumina (NEB) and sequenced on an Illumina NextSeq 500 platform. SMRT sequencing was performed at Wuhan Institute of Biotechnology, China. De novo assemblies of PacBio RSII reads and Illumina reads were performed by the hierarchical genome assembly process (HGAP, Paciﬁc Biosciences) and the CLC Genomics Workbench (CLC bio, Denmark) respectively. Long assembled contigs obtained from PacBio reads were used to align and join the contigs obtained from the Illumina assembly results. The completed plasmid sequence was confirmed by PCR and then annotated with the RAST tool [34] and the NCBI Prokaryotic Genome Annotation Pipeline (PGAP). Each plasmid was sequenced using both Illumina and PacBio platforms and only high-quality data were used for further analysis.

### *Salmonella* infection and treatment model

The LD50 of the *Salmonella* strains tested in this study was first determined in a mouse model. Exponentially growing organisms were centrifuged, washed twice and resupended with sterile saline to achieve an OD value of 0.5. Three to 4-week-old male NIH mice were randomly separated into 5 groups, with 5 mice per group. Each group was injected with the following doses of *Salmonella* through the tail vein: 0, 5 × 10E4, 5 × 10E5, 5 × 10E6 and 5 × 10E7 CFU. Number of dead mice was recorded every 12 h till 48 h, then the LD50 at different time courses was determined and the dose of LD50 would be used for infecting in treatment experiments. For treatment experiments, inoculation *Salmonella* strains PY1 and PY1-TC were prepared as described above; 3–4-week-old male NIH mice were randomly separated into several groups and infected with ∼1 × 10E6 of bacteria by intravenous injection. Infections were allowed to establish for 1 h prior to treatment with 15 mg/kg ciprofloxacin and 20 mg/kg ceftiofur, respectively, with the therapeutic dose for clinical treatment of *Salmonella* infection. The rationale for use of ceftiofur instead of ceftriaxone is that ceftiofur is a veterinary drug with similar properties to those of ceftriaxone; both belong to the third generation of cephalosporin. Both drugs have similar MICs when tested against Sa44. Saline treatment was used as a control to evaluate the therapeutic effect. As the ciprofloxacin was **less** effective in therapy compare with ceftiofur, half of the number of bacteria was used to infect mice once time, and the same method with ciprofloxacin was used for treatment as described above. The death number of the mice was recorded every 12 h till 96 h post inoculation. The efficacy of antibiotic treatment in different groups of mice was calculated.

All experimental mice were obtained from the Guangdong Provincial Laboratory Animal Center, Guangzhou, China and housed in individually ventilated cage (IVC) with free access to food and water. All experiments were conducted strictly in accordance with the guidelines of the Chinese Association for the Accreditation of Laboratory Animals Care (CAALAC), including the relevant local animal welfare regulatory bodies in China. The animal works were approved by the animal ethics committee of South China Agriculture University, Guangzhou, China.

## Authors’ contributions

KCC performed the experiments; EWCC contributed to study design and manuscript writing; SC designed the study, wrote the manuscript and supervised the whole project.

## Data Availability

The sequencing data of the plasmids have been deposited in GenBank under the accession numbers pSa44-CIP-CRO (MH430881), pSa44-CIP (MH430882) and pSa44-CRO (MH430883).

## References

[1] Gomez TM, Motarjemi Y, Miyagawa S, et al. Foodborne salmonellosis. World Health Stat Q. 1997;50:81–89. 9282390

[2] Hohmann EL. Nontyphoidal salmonellosis. Clin Infect Dis. 2001;32:263–269. DOI:10.1086/318457. 11170916

[3] Multidrug-resistant Salmonella serotype Typhimurium–United States, 1996 MMWR (Morbidity and Mortality Weekly Report). 1997, vol. 46, p. 308-310. 9132584

[4] Markogiannakis A, Tassios PT, Lambiri M, et al. Multiple clones within multidrug-resistant Salmonella enterica serotype Typhimurium phage type DT104. The Greek Nontyphoidal Salmonella Study Group. J Clin Microbiol. 2000;38:1269–1271. 10699039 10.1128/jcm.38.3.1269-1271.2000 PMC88604

[5] Glynn MK, Bopp C, Dewitt W, et al. Emergence of multidrug-resistant *Salmonella enterica* serotype typhimurium DT104 infections in the United States. N Engl J Med. 1998;338:1333–1339. DOI:10.1056/NEJM199805073381901. 9571252

[6] Leekitcharoenphon P, Hendriksen RS, Le Hello S, et al. Global genomic epidemiology of *Salmonella enterica* Serovar Typhimurium DT104. Appl Environ Microbiol. 2016;82:2516–2526. DOI:10.1128/AEM.03821-15. 26944846 PMC4959494

[7] Zhao S, Blickenstaff K, Glenn A, et al. beta-Lactam resistance in salmonella strains isolated from retail meats in the United States by the National Antimicrobial Resistance Monitoring System between 2002 and 2006. Appl Environ Microbiol. 2009;75:7624–7630. DOI:10.1128/AEM.01158-09. 19854922 PMC2794113

[8] NARMS National Antimicrobial Resistance Monitoring System (NARMS): 2014 Human isolates surveillance report; 2014. http://www.cdc.gov/narms/pdf/2014-annual-report-narms-508c.pdf.

[9] Wong MH, Yan M, Chan EW, et al. Emergence of clinical *Salmonella enterica* serovar Typhimurium isolates with concurrent resistance to ciprofloxacin, ceftriaxone, and azithromycin. Antimicrob Agents Chemother. 2014;58:3752–3756. DOI:10.1128/AAC.02770-13. 24752251 PMC4068579

[10] Ho P, Lo WU, Yeung MK, et al. Dissemination of pHK01-like incompatibility group IncFII plasmids encoding CTX-M-14 in Escherichia coli from human and animal sources. Vet Microbiol. 2012;158:172–179. doi: 10.1016/j.vetmic.2012.02.004 22386670

[11] Hooper DC. Emerging mechanisms of fluoroquinolone resistance. Emerg Infect Dis. 2001;7:337–341. DOI:10.3201/eid0702.700337 11294736 PMC2631735

[12] Chen S, Zhao S, White DG, et al. Characterization of multiple-antimicrobial-resistant *Salmonella* serovars isolated from retail meats. Appl Environ Microbiol. 2004;70:1–7. doi: 10.1128/AEM.70.1.1-7.2004 14711619 PMC321239

[13] Szmolka A, Fortini D, Villa L, et al. First report on IncN plasmid-mediated quinolone resistance gene qnrS1 in porcine *Escherichia coli* in Europe. Microb Drug Resist. 2011;17:567–573. DOI:10.1089/mdr.2011.0068. 21834664

[14] Gunell M, Webber MA, Kotilainen P, et al. Mechanisms of resistance in nontyphoidal *Salmonella enterica* strains exhibiting a nonclassical quinolone resistance phenotype. Antimicrob Agents Chemother. 2009;53:3832–3836. DOI:10.1128/AAC.00121-09. 19596880 PMC2737843

[15] Ferrari R, Galiana A, Cremades R, et al. Plasmid-mediated quinolone resistance by genes qnrA1 and qnrB19 in Salmonella strains isolated in Brazil. J Infect Dev Ctries. 2011;5:496–498. doi: 10.3855/jidc.1735 21727652

[16] Ceyssens PJ, Mattheus W, Vanhoof R, et al. Trends in serotype distribution and antimicrobial susceptibility in *Salmonella enterica* isolates from humans in Belgium, 2009 to 2013. Antimicrob Agents Chemother. 2015;59:544–552. DOI:10.1128/AAC.04203-14. 25385108 PMC4291397

[17] Abgottspon H, Zurfluh K, Nuesch-Inderbinen M, et al. Quinolone resistance mechanisms in Salmonella enterica serovars Hadar, Kentucky, Virchow, Schwarzengrund, and 4,5,12:i:-, isolated from humans in Switzerland, and identification of a novel qnrD variant, qnrD2, in S. Hadar. Antimicrob Agents Chemother. 2014;58:3560–3563. DOI:10.1128/AAC.02404-14. 24733466 PMC4068464

[18] Nuesch-Inderbinen M, Abgottspon H, Sagesser G, et al. Antimicrobial susceptibility of travel-related Salmonella enterica serovar Typhi isolates detected in Switzerland (2002-2013) and molecular characterization of quinolone resistant isolates. BMC Infect Dis. 2015;15:212 DOI:10.1186/s12879-015-0948-2. 25963025 PMC4435775

[19] Wong MH, Chan EW, Liu LZ, et al. PMQR genes oqxAB and aac(6’)Ib-cr accelerate the development of fluoroquinolone resistance in Salmonella typhimurium. Front Microbiol. 2014;5:521 DOI:10.3389/fmicb.2014.00521. 25324840 PMC4183184

[20] Lin D, Chen K, Wai-Chi Chan E, et al. Increasing prevalence of ciprofloxacin-resistant food-borne *Salmonella* strains harboring multiple PMQR elements but not target gene mutations. Sci Rep. 2015;5:14754 DOI:10.1038/srep14754. 26435519 PMC4648336

[21] Li R, Lin D, Chen K, et al. First detection of AmpC beta-lactamase bla(CMY-2) on a conjugative IncA/C plasmid in a Vibrio parahaemolyticus isolate of food origin. Antimicrob Agents Chemother. 2015;59:4106–4111. DOI:10.1128/AAC.05008-14. 25918142 PMC4468700

[22] Chen K, Dong N, Zhao S, et al. Identification and characterization of conjugative plasmids that encode ciprofloxacin resistance in Salmonella. Antimicrob Agents Chemother. 2018;62:00575–00518. 10.1128/AAC.00575-18 PMC6105805 29760137

[23] Wang Y, Zhang R, Li J, et al. Comprehensive resistome analysis reveals the prevalence of NDM and MCR-1 in Chinese poultry production. Nat Microbiol. 2017;2:16260. doi: 10.1038/nmicrobiol.2016.260 28165472

[24] Chen S, Cui S, McDermott PF, et al. Contribution of target gene mutations and efflux to decreased susceptibility of *Salmonella enterica* serovar Typhimurium to fluoroquinolones and other antimicrobials. Antimicrob Agents Chemother. 2007;51:535–542. DOI:10.1128/AAC.00600-06. 17043131 PMC1797773

[25] Kim J, Han X, Bae J, et al. Prevalence of plasmid-mediated quinolone resistance (PMQR) genes in non-typhoidal Salmonella strains with resistance and reduced susceptibility to fluoroquinolones from human clinical cases in Alberta, Canada, 2009-13. J Antimicrob Chemother. 2016;71:2988–2990. DOI:10.1093/jac/dkw232. 27342547

[26] Ferrari R, Galiana A, Cremades R, et al. Plasmid-mediated quinolone resistance (PMQR) and mutations in the topoisomerase genes of *Salmonella enterica* strains from Brazil. Braz J Microbiol. 2013;44:657–662. DOI:10.1590/S1517-83822013000200046. 24294265 PMC3833171

[27] Pribul BR, Festivo ML, Rodrigues MS, et al. Characteristics of quinolone resistance in *Salmonella* spp. isolates from the food chain in Brazil. Front Microbiol. 2017;8:299, DOI:10.3389/fmicb.2017.00299. 28352250 PMC5348486

[28] Wong MH-Y, Chan EW-C, Chen S. IS 26-mediated formation of a virulence and resistance plasmid in *Salmonella enteritidis*. J Antimicrob Chemother. 2017;72:2750–2754. doi: 10.1093/jac/dkx238 29091201

[29] Xie M, Li R, Liu Z, et al. Recombination of plasmids in a carbapenem-resistant NDM-5-producing clinical *Escherichia coli* isolate. J Antimicrob Chemother. 2018;73:1230–1234. doi: 10.1093/jac/dkx540 29373691

[30] Garcia A, Navarro F, Mirã E, et al. Acquisition and diffusion of bla CTX-M-9 gene by R478-IncHI2 derivative plasmids. FEMS Microbiol Lett. 2007;271:71–77. DOI:10.1111/j.1574-6968.2007.00695.x. 17391369

[31] Meyer R. Replication and conjugative mobilization of broad host-range IncQ plasmids. Plasmid. 2009;62:57–70. DOI:10.1016/j.plasmid.2009.05.001. 19465049 PMC2752045

[32] Sun J, Yang R-S, Zhang Q, et al. Co-transfer of blaNDM-5 and mcr-1 by an IncX3-X4 hybrid plasmid in Escherichia coli. Nat Microbiol. 2016;1:16176 DOI:10.1038/nmicrobiol.2016.176. 27668643

[33] CLSI Performance standards for antimicrobial susceptibility testing, twenty-sixth informational supplement. CLSI document M100-S26. Wayne, PA: Clinical and Laboratory Standards Institute; 2016.

[34] Overbeek R, Olson R, Pusch GD, et al. The SEED and the rapid annotation of microbial genomes using subsystems technology (RAST). Nucleic Acids Res. 2014;42:D206–D214. DOI:10.1093/nar/gkt1226. 24293654 PMC3965101

